# Trehalose Alleviated Salt Stress in Tomato by Regulating ROS Metabolism, Photosynthesis, Osmolyte Synthesis, and Trehalose Metabolic Pathways

**DOI:** 10.3389/fpls.2022.772948

**Published:** 2022-03-11

**Authors:** Yan Yang, Yandong Yao, Jing Li, Jing Zhang, Xiaodan Zhang, Lixia Hu, Dongxia Ding, Emily Patience Bakpa, Jianming Xie

**Affiliations:** College of Horticulture, Gansu Agricultural University, Lanzhou, China

**Keywords:** trehalose, salt stress, osmotic substances, carbohydrates, trehalose metabolism, antioxidant system

## Abstract

Trehalose plays a critical role in plant response to salinity but the involved regulatory mechanisms remain obscure. Here, this study explored the mechanism of exogenous trehalose-induced salt tolerance in tomato plants by the hydroponic test method. Our results indicated that 10 mM trehalose displayed remarkable plant biomass by improving growth physiology, which were supported by the results of chlorophyll fluorescence and rapid light–response curve. In the salinity environment, trehalose + NaCl treatment could greatly inhibit the decrease of malondialdehyde level, and it increases the contents of other osmotic substances, carbohydrates, K^+^, and K^+^/Na^+^ ratio. Meanwhile, trehalose still had similar effects after recovery from salt stress. Furthermore, trehalose pretreatment promoted trehalose metabolism; significantly increased the enzymatic activity of the trehalose metabolic pathway, including trehalose-6-phosphate synthase (TPS), trehalose-6-phosphate phosphatase (TPP), and trehalase (TRE); and upregulated the expression of *SlTPS1*, *SlTPS5*, *SlTPS7*, *SlTPPJ*, *SlTPPH*, and *SlTRE* under saline conditions. However, the transcriptional levels of *SlTPS1*, *SlTPS5*, and *SlTPS7* genes and the activity of TPS enzyme were reversed after recovery. In addition, we found that hydrogen peroxide (H_2_O_2_) and superoxide anion (O_2_^−^) were accumulated in tomato leaves because of salt stress, but these parameters were all recovered by foliar-applied trehalose, and its visualization degree was correspondingly reduced. Antioxidant enzyme activities (SOD, POD, and CAT) and related gene expression (*SlCu/Zn-SOD*, *SlFe-SOD*, *SlMn-SOD*, *SlPOD*, and *SlCAT*) in salt-stressed tomato leaves were also elevated by trehalose to counteract salt stress. Collectively, exogenous trehalose appeared to be the effective treatment in counteracting the negative effects of salt stress.

## Introduction

Salt stress is a severe environmental pressure affecting crop yields worldwide (Yu et al., 2020). It is regarded as a contemporary agricultural issue restricting land use and a major problem limiting the increase in demand for food crops (Abbasi et al., 2016; Isayenkov and Maathuis, 2019). At present, about 20% of the world’s arable land and nearly half of the irrigated land are affected by salt toxicity (Roychoudhury et al., 2013). When the salt in the external environment exceeds the salinity threshold of plants, high salt content and long action time cause irreversible changes and response on all functional levels of plant organisms and even lead to plant death (Yue et al., 2012; Zhang et al., 2016). Salt stress mainly induced ion toxicity, which subsequently provoked osmotic and oxidative stress (Tahjib-Ul-Arif et al., 2018). Therefore, to minimize the toxic effects of salt stress, plants have evolved various defense strategies, such as scavenging reactive oxygen species (ROS) through various antioxidant enzymes, non-enzymatic antioxidants, free amino acids and soluble phenols, and using the accumulated total and reducing sugars (e.g., trehalose) could stabilize cell membranes (Farooq et al., 2017). Nowadays, adopting grafting (Penella et al., 2017; Singh et al., 2020), crop rotation (Ahmad et al., 2013; Neves et al., 2015) and other agricultural operations, selecting salt-tolerant varieties (Tran et al., 2021), and applying exogenous substances (Zheng et al., 2016; Ali et al., 2020) are the most simple and common methods to improve the salt tolerance of plants and overcome the problem of soil salinization in production.

Trehalose, a non-reducing disaccharide, was first discovered by Wiggers and others from ergot in rye in the 19th century, which widely existed in organisms (Kosar et al., 2019). In addition, Bianchi et al. (1993) found that only certain drought-tolerant plants, such as *Myrothamnus flabellifolius*, accumulate trehalose sufficiently to act as an osmolyte. Given the low content of trehalose in plants, it is more used as a signal molecule, and it plays a role in the regulation of plant growth, development, and metabolism in response to extreme environment such as high temperature, salinity, drought, and cold stress (Fernandez et al., 2010; Tournu et al., 2013; Henry et al., 2015; Fichtner and Lunn, 2021). Trehalose 6-phosphate (T6P) was considered to be a signaling metabolite communicating the carbohydrate state of plants to other pathways involved in growth, development and responses to the environment (Fichtner et al., 2021). Therefore, the various metabolic functions of trehalose may be caused by its role in sugar signal transduction.

Trehalose has only one metabolic pathway (TPS-TPP) in plants, which is synthesized *via* the intermediate T6P (Lin et al., 2017). At present, the role of trehalose metabolism in improving stress-tolerant plants has received considerable interest. Recent studies have shown that modification of trehalose pathway in transgenic plants was associated with stress tolerance and recovery. In addition, exogenous application of trehalose or T6P also showed promising prospects in crop improvement. For example, low concentration of exogenous trehalose can reduce the accumulation of sodium ions in plants, whereas high concentration of trehalose can prevent the loss of chlorophyll in leaves and root damage caused by high salt, thereby reducing the damage caused by salt ions to plants (Lunn et al., 2014; Islam et al., 2019). In addition, studies have reported that exogenous trehalose greatly alleviates ion imbalance, ROS outbreak, and programmed cell death caused by salt stress in *Arabidopsis thaliana* seedlings (Garapati et al., 2015), maize (Henry et al., 2015), *Catharanthus roseus* (Chang et al., 2014), and tomato (Feng et al., 2019). Furthermore, the molecular mechanism revealed that salt stress could induce *OsTPP1* gene expression in rice (Zhang et al., 2017), but *AtTPPD*-overexpressed plants were more tolerant to high salt stress (Krasensky et al., 2014).

Tomato is a main fruit and vegetable of worldwide. However, the regulatory mechanism of trehalose on the trehalose metabolism pathway and oxidative damage under salt stress has not been evaluated in tomato. In this study, “Micro-Tom” was used as the material to study the effects of trehalose application on the growth physiology, photosynthesis, osmotic regulation, carbohydrate, trehalose metabolism, and antioxidant systems of tomato plants under salt stress. Furthermore, this study proposed that trehalose could be used as a potential technology to improve plant culture in salinized areas, so as to cope with sustainable agricultural development in the era of climate change, and provided a new physiological basis for further dissection of the mechanism of trehalose mediated salt resistance in tomato seedlings.

## Materials and Methods

### Experimental Materials and Growing Conditions

The tomato seeds (Micro-Tom) were soaked at 50°C–55°C for 15 min and then rinsed with distilled water 3–5 times. The seeds were placed on a moist filter paper and kept in the dark to germinate at 28°C± 1°C for 3 days. The uniformly germinated tomato seeds were sown in pots filled with vermiculite and perlite substrate (vermiculite:perlite = 3:1). When the first true leaf of tomato seedlings appeared, uniform seedlings were transferred to opaque containers, cultured with half concentration of whole Hoagland’s nutrient solution, and kept at the same conditions with a light intensity of 360 μmol m^−2^ s^−1^, temperature of 26 ± 1°C/20 ± 1°C, photoperiod 16/8 h, and relative humidity of 60%. Seedlings were replaced with whole Hoagland’s nutrient solution after 5 days. Afterward, the nutrient solution was changed at 5-day intervals. The experiments were developed using consistent seedlings with four leaves.

### Experimental Design

#### NaCl Concentration

Five concentration gradients of NaCl were set in this experiment, including 0 mM NaCl (CK), 50 mM NaCl (S1), 100 mM NaCl (S2), 150 mM NaCl (S3), and 200 mM NaCl (S4). Different concentrations of sodium chloride were added into Hoagland nutrient solution. After 5-day treatment, the morphology of the plants was observed, and the physiological indexes were measured to select the moderate NaCl concentration. Each treatment was repeated three times with 120 seedlings.

#### Trehalose Concentration

Four trehalose concentrations were set as follows Hoagland nutrient solution (CK), NaCl (T0), 5 mM trehalose + NaCl (T1), 10 mM trehalose + NaCl (T2), and 25 mM trehalose + NaCl (T3). The NaCl concentration was selected by the above experiments. Different trehalose solutions (equal volumes of pure water for CK and T0) were thoroughly sprayed on both sides of the leaves before NaCl treatment for two consecutive days. After 5 days of NaCl treatment, these seedlings were collected for assays of phenotypic changes and physiological parameters.

#### Alleviating Effect of Exogenous Trehalose on Tomato Under Salt Stress

On the basis of the above experiments, four treatments were set Hoagland nutrient solution (CK), trehalose (T), NaCl (S), and trehalose + NaCl (S + T). The spraying method of trehalose was the same as above experiment (equal volumes of pure water for CK and S). The NaCl treatment time was 5 days, and all treatments were replaced with Hoagland nutrient solution for another 5 days after the NaCl treatment. Functional leaves (fully spreading the functional leaves from the bottom of the plant to the second and third branches) were collected from four treatments at different time points under salt and wipe-salt periods. Before salt stress treatment was recorded as 0 day, the stress and recovery periods were marked as X and H, respectively.

### Morphological Index Determination

Plant height, stem diameter, and fresh and dry weight of shoots and roots were measured after treatment for 5 days. Plant height was determined by measuring the length from the bottom of the stem to the top of the growing point; stem diameter was measured using a vernier caliper, and the whole seedlings were collected and rinsed with deionized water, surface dried and separated into roots and shoots to obtain fresh weight, and then dried to constant weight at 80°C to obtain dry weight.

### Root Analysis

Root images were scanned with the Epson Expression 11000XL. Total root length, root surface area, root volume, root tips, and forks were calculated using Root analysis software Win Rhizo 5.0.

### Plasma Membrane Permeability

The relative electrical conductivity (REC) and cell membrane injury rate were examined according to Zhou et al. (2013).

### Chlorophyll Content and Chlorophyll Fluorescence Parameters

A total of 80% acetone was used to determine chlorophyll content referring to Lichtenthaler (1987) with some modifications. The chlorophyll fluorescence parameters of tomato leaves were measured using the Maxi Imaging PAM chlorophyll fluorescence apparatus referring to Hu et al. (2017) with some modifications (Walz, Effeltrich, Germany). After 30 min of dark adaptation, the reaction center of the tested plants was completely open. The operating parameters of the instrument were as follows the detection light intensity was 0.1 μmol m^−2^ s^−1^; the actinic light intensity was 111 μmol m^−2^ s^−1^; the saturation pulse light intensity was 2700 μmol m^−2^ s^−1^; the pulse light time was 0.8 s, and the time interval was 20 s. In the absence of actinic light, the initial fluorescence (Fo) and maximum fluorescence (Fm) under darkness were measured by the saturation pulse light. Then, the kinetics curve of chlorophyll fluorescence was determined. Steady-state fluorescence (Fs) was obtained by stimulating normal photosynthesis for 5 min with actinic light (Al, 81 μmol m^−2^ s^−1^). In addition, the maximum fluorescence yield (Fm′) was obtained after 0.8 s of saturated pulse light irradiation. When photochemical light was turned off and far-red light was turned on, initial fluorescence under light (Fo′) was measured.


Fv/Fm=(Fm−Fo)/Fm

Fv'/Fm'=Fm'−Fo'/Fm'

YII=Fm'−Fs/Fm'

qP=Fm'−Fs/Fm'−Fo'

qN=Fm−Fm'/Fm−Fo'

Determination of rapid light curve: in the light curve window of Maxi Imaging PAM, the gradients of photosynthetical active radiation (PAR) intensity were set as 0, 2, 22, 57, 112, 187, 282, 397, 532, 702, 927, and 1,252 μmol m^−2^ s^−1^. The relative electron transfer rate of PSII was measured with the increase of light intensity PAR. The relative electron transport rate is shown as follows: $\mathrm{rETR}=\left({\mathrm{Fm}}^{\prime }-\mathrm{Ft}\right)/\mathrm{Fm}\prime \times \mathrm{Par}\times 0.5\times 0.84$ , where Fm′ represents the maximum fluorescence yield under light adaptation; Ft represents the instantaneous fluorescence yield; Par represents the photosynthetically active radiation intensity; 0.5 represents the light absorbed by the plant, which is equally distributed between the two photosystems; 0.84 represents the light absorption coefficient. The light–response curve was fitted by the following model equation: P=Pm×(1−exp(−α×Par/Pm))×exp(−β×Par/Pm) , where Pm represents the maximum relative electron transfer rate, rETRmax; *α* represents the initial slope of the rapid light curve; *β* represents the photoinhibition parameter; Ik ( Ik=Pm/α ) represents half saturation and light intensity. The value of Ik was two times that of the saturated light intensity (Ek).

### Osmotic Regulation Substances

The free proline (Pro) content was assayed as described by Ma et al. (2015) with some modifications. Malondialdehyde (MDA) content estimation was conducted following the method described by Platt et al. (1981). The hydrochloric acid–methanol method was used to measure the total phenol content (Vlaic et al., 2018). Total flavonoid in tomato leaves was appraised following the method of sodium nitrite–aluminum nitrate (Hand et al., 2017). The test kit (Michy Biomedical, Suzhou, China) was used to determine the content of glycine betaine (GB). The method described by Wang et al. (2020a) was used to quantify the soluble protein content.

### Carbohydrate Contents and Trehalose Metabolism

Extraction of carbohydrate: fresh leaf samples (0.5 g) of each replicate were homogenized by adding 5 ml of 80% ethanol. Ultrasonic extraction was performed at 35°C for 20 min. Then, the solution was centrifuged at 12,000 rpm/min for 15 min, and the supernatant was separated. Afterward, 2 ml of 80% ethanol was added to the precipitate, and the above-mentioned operation was repeated two times. Finally, the final volume was increased to 10 ml by adding 80% ethanol. The extract was evaporated and dried in a rotary evaporator (60°C) and dissolved with 1 ml of ultra-pure water and 1 ml of acetonitrile. After filtration using a 0.22-μm millipore filter, the solution was tested on the machine.

Analysis conditions and parameters of high-performance liquid chromatography mass spectrometry: Chromatograph: Aglient1100 high-performance liquid chromatograph. Chromatographic column: Xbridge^®^ BEH™ Amide 2.5 μm. Mobile phase: 75% acetonitrile + 0.2% triethylamine + 24.8% ultrapure water. Flow rate: 0.8 ml/min. Sample size: 10 μl. Detection wavelength: 254 nm. Column temperature: 40°C.

Standard curve drawing: Standard samples of glucose, fructose, and sucrose (Sigma, purity ≥99.9%) were accurately weighed and prepared into mother liquor of 10, 10, and 10 mg/ml, respectively. Glucose, fructose, and sucrose were absorbed (1 ml each) and prepared into a mixed standard. Then, the mixed standard was diluted into eight standard solutions with different mass concentrations of 0.1, 0.25, 0.5, 0.75, 1, 1.5, 2, and 2.5 mg/ml. Based on the above-mentioned chromatographic conditions, the standard curve of different sugars with mass concentration as the abscissa and chromatographic peak area as the ordinate was drawn.

The trehalose content was measured using a trehalose kit (Comin Botechnology, Suzhou, China). The test kit (Michy Biomedical, Suzhou, China) was used to determine the activity of trehalose-6-phosphate phosphatase (TPP), trehalose-6-phosphate synthase (TPS), and trehalase (TRE).

### ROS Metabolism Assay

The contents of hydrogen peroxide (H_2_O_2_) and superoxide anion (O_2_^−^) were measured by the H_2_O_2_ and O_2_^−^ assay kit (Comin Botechnology, Suzhou, China). H_2_O_2_ and O_2_^−^ were visually detected in the fourth leaves according to the method of Tang et al. (2021). The activity of superoxide dismutase (SOD), peroxidase (POD), and catalase (CAT) were determined in tomato leaves using the SOD, POD, and CAT assay kit (Comin Botechnology, Suzhou, China). In brief, SOD removed O_2_^−^ and inhibited the formation of formazan; thus, SOD was calculated by measuring the formazan content at 450 nm. POD was estimated by monitoring the increase in absorbance at 470 nm when guaiacol was oxidized by H_2_O_2_ to release O_2_. CAT was measured by monitoring the absorbance value of H_2_O_2_ oxidation product (H_2_MoO_4_·*x*H_2_O)*_n_* at 405 nm.

### Ion Contents

The contents of Na^+^ and K^+^ were measured according to the method of Li et al. (2020) with modifications. Simply put, fresh leaves were dried in an oven. The dried sample (0.5 g) was incubated overnight with 5 ml of concentrated sulfuric acid in a conical flask. Whereafter, the sample was digested with H_2_O_2_ until it was clear and transparent, after which the cooled digestive solution was diluted to 50 ml with distilled water. Na^+^ and K^+^ contents were determined by atomic absorption spectrometer (ZEEnit 700P, Analytik Jena, Germany).

### Total RNA Extraction and Reverse Transcription Polymerase Chain Reaction Analysis

Total RNA from tomato leaves was extracted using the RNA extraction kit (Accyrate Biotechnology Co., Ltd., China). The extracted RNA samples were reverse-transcribed using the EVO-MLV reverse transcription kit (Accyrate Biotechnology Co., Ltd., China) to obtain cDNA. The SYBR Green Premix Pro Taq HS qPCR Kit II (Accyrate Biotechnology Co., Ltd., China) was used for RT-PCR. Using tomato actin as the internal reference gene, the gene bank entry number used to design the primer sequence is shown in Table 1. The reaction system was 20 μl: 1 μl of cDNA, 2 μl of upstream and downstream primers, 10 μl of SYBR, and 7 μl of ddH_2_O. Real-time PCR was performed using the Light Cycler^®^ 96 Real-Time PCR System (Roche, Switzerland). The relative expression level of mRNA was calculated using the 2^−ΔΔCT^ method.

**Table 1:** Primer sequences used for qRT-PCR.

Gene name	Sequence (5′-3′)	GenBank accession number
Actin	F: AATGAACTTCGTGTGGCTCCAGAG	NC_015447.3
	R: ATGGCAGGGGTGTTGAAGGTTTC	
*SlTPPJ*	F: TGAGATGAGGGAAGCAGTAAGAAACAC	XM_004237846.4
	R: CAACACGACGGAAATGTACGGATAAAC	
*SlTPPH*	F: ATGTTCGTGGTGGAGATGGTTTCG	XM_010323164.3
	R: ATCAGGGTCATCAACAATGGGAGAAAG	
*SlTPS1*	F: AATGGAGGACGACGAAAGAGTAGATTG	NM_001246967.2
	R: GAAGAGACGATATGGGCTACCGAAAG	
*SlTPS5*	F: GACAAGATGGTGAGGAAGGATGGAAC	XM_010327261.3
	R: CTGGAGATAGCGGAAGCATGTAGTG	
*SlTPS7*	F: GTGCTGTGCGTAGGTGATGATAGG	XM_010318541.2
	R: TCTGATGGAGGAGGAGGAATGGATTC	
*SlTRE*	F: AAGATCCCTTCCTGCTTTGCTCAAG	XM_004245430.4
	R: GTCAGATCAGATCCGTTGCTCATCC	
*SlCu/Zn-SOD*	F: TCACCACAACCAGCACTACCAATTC	NM_001247840
	R: GAAGTCCAGGAGCAAGTCCAGTTATAC	
*SlFe-SOD*	F: CCGCCACTGCCTCTGCTAATTC	NM_001246860
	R: GACATACGCCCTGTGATGCTTCC	
*SlMn-SOD*	F: CCACCACCAGAAGCATCATCAGAC	XM_001330621
	R: GTCAATAGCCCAGCCAAGAGAACC	
*SlPOD*	F: GCTAGAGATGCAGTTGTGGCTACG	NM_001302921
	R: ATGATGAGCAACGAGACACTCCAATAG	
*SlCAT*	F: GCTCCCAGTTAATGCTCCCAAGTG	NM_001247898
	R: GACTCGTGGATCGGATAAAGACTCAAC	

### Statistical Analysis

All experiments were performed in triplicate, and the results were expressed as mean ± standard error. ANOVA was performed using SPSS 22.0 (SPSS Institute Inc., United States), and Duncan’s multiple range test with a probability level of 0.05 was used for comparison. All data were compiled by OriginPro 2017 (OriginLab Institute Inc., United States).

## Results

### Fluctuation of Salinity Affected Physiology and Root Characteristics

**Figure 1:**
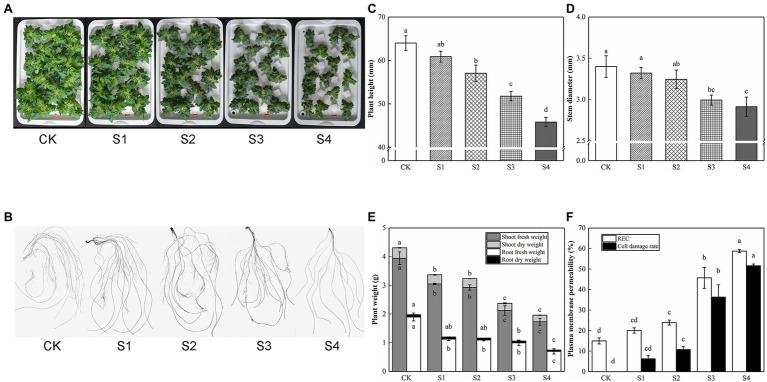
The growth and physiological indices affected by salinity in tomato plants. Photographs and data of tomato plants under saline condition were obtained after 5 days. **(A)** Morphological changes. **(B)** Root performance. **(C)** Plant height. **(D)** Stem diameter. **(E)** Plant weight. **(F)** Plasma membrane permeability. The results showed the mean ± SE of three replicates, and the different letters denote the significant difference among treatments (*p* < 0.05), according to Duncan’s multiple test. CK, control; S1, 50 mM NaCl; S2, 100 mM NaCl; S3, 150 mM NaCl; S4, 200 mM NaCl.

With the increase of salt stress concentration, the inhibitory effect of tomato seedling growth strengthened gradually; the degree of leaf edge curl and damage increased slightly, and the root length, root surface area, root volume, root tips, and forks were slowly decreased (Figure 1; Table 2). REC was considered as an important indicator of cell membrane permeability. Salt stress increased REC and cell damage rate, which was consistent with the damage degree of appearance morphology. Compared with CK, no significant discrepancies were observed in plant height, stem diameter, plant weight, REC, and cell damage rate in S1; plant height, stem diameter, shoot fresh weight, root fresh weight, and shoot dry weight in plants fed with S2 were significantly decreased by 10.85%, 4.59%, 25.86%, 42.34%, and 15.60%, respectively, but the difference in root dry weight was not significant, which was classified as mild stress. Compared with S2, S3 had lower plant height, stem diameter, and plant weight, which was defined as moderate stress. Herein, the plant height, stem diameter, and plant weight of S4 were the lowest, which was identified as severe stress. Therefore, 150 mM of NaCl treatment (S3) was used as a moderate concentration of salt stress in subsequent experiments.

**Table 2:** The root parameters affected by salinity in tomato plants.

Treatment	Total root length (cm)	Surface surface area (cm^2^)	Volume (cm^3^)	Tips number	Forks
CK	1046.189 ± 14.9a	132.414 ± 6.234a	1.906 ± 0.04a	1483.667 ± 221.446a	2062.333 ± 181.23a
S1	1032.018 ± 81.777a	114.995 ± 3.84ab	1.024 ± 0.016bc	1003.333 ± 57.138b	1762.667 ± 283.705a
S2	794.974 ± 66.984b	107.633 ± 8.759ab	1.16 ± 0.093b	883.667 ± 111.459bc	1,035 ± 182.138b
S3	672.238 ± 51.318bc	100.139 ± 8.566b	0.89 ± 0.081bc	600.333 ± 28.298c	990 ± 145.812b
S4	486.455 ± 62.087c	64.571 ± 9.971c	0.709 ± 0.187c	509.667 ± 84.548c	672.667 ± 142.612b

Data of tomato plants under saline condition were obtained after 5 days. Values (mean ± SE) indicated the average of three independent experiments. Values not sharing the same letters in the same list indicated statistical significance by Duncan’s multiple range test at 5% level. CK, control; S1, 50 mM NaCl; S2, 100 mM NaCl; S3, 150 mM NaCl; S4, 200 mM NaCl.

### Trehalose Concentrations Affected the Physiology, Chlorophyll Fluorescence, Light–Response Curve

**Figure 2:**
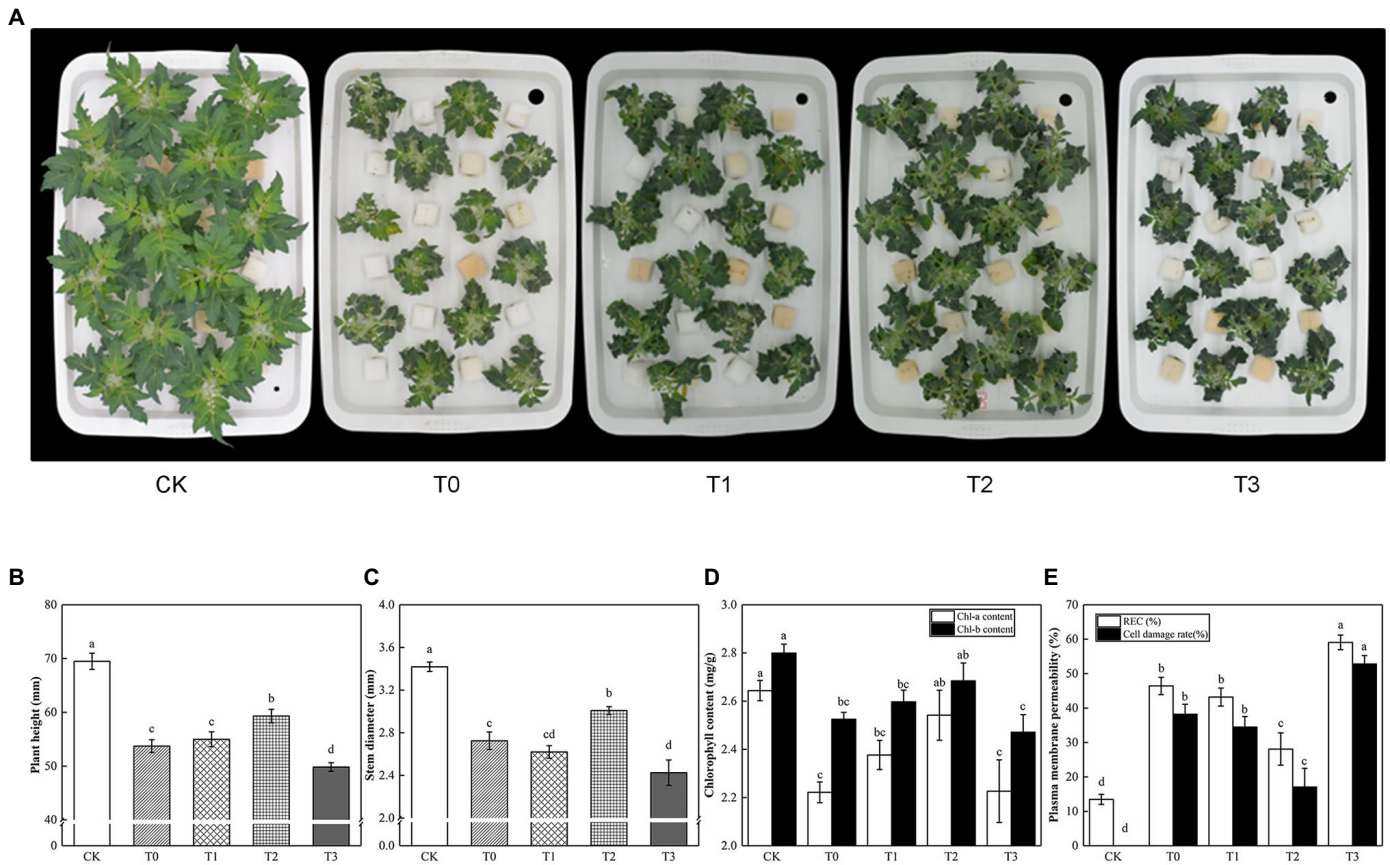
Effect of different concentrations of trehalose on the growth and physiological parameter of tomato seedlings. Photographs and data were obtained after 5 days of treatment. **(A)** Morphological changes. **(B)** Plant height. **(C)** Stem diameter. **(D)** Chlorophyll content. **(E)** Plasma membrane permeability. The results showed the mean ± SE of three replicates, and the different letters denote the significant difference among treatments (*p* < 0.05), according to Duncan’s multiple test. CK, control; T0, 150 mM NaCl; T1, 150 mM NaCl +5 mM trehalose; T2, 150 mM NaCl +10 mM trehalose; T3, 150 mM NaCl +25 mM trehalose.

Under saline conditions, exogenous trehalose had a dose-dependent effect on tomato seedlings (Figure 2). With the increase of trehalose dosage, the plant height and stem diameter of tomato initially showed an increasing trend and then a reducing trend. In our study, we reported that most treatments with trehalose increased plant height and stem diameter, and T2 was the most significant. As shown in Figure 2D, T2 alleviated the symptoms of leaf chlorosis caused by salt stress. When compared with T0, the chlorophyll of T1 and T3 had no significant difference. Meanwhile, the REC and cell injury rates of T2 were lower by 28.08% and 17.07%, respectively, compared with T0. Hence, we found that T2 conducted the most distinct anti-senescence phenotype in tomato plants.

**Figure 3:**
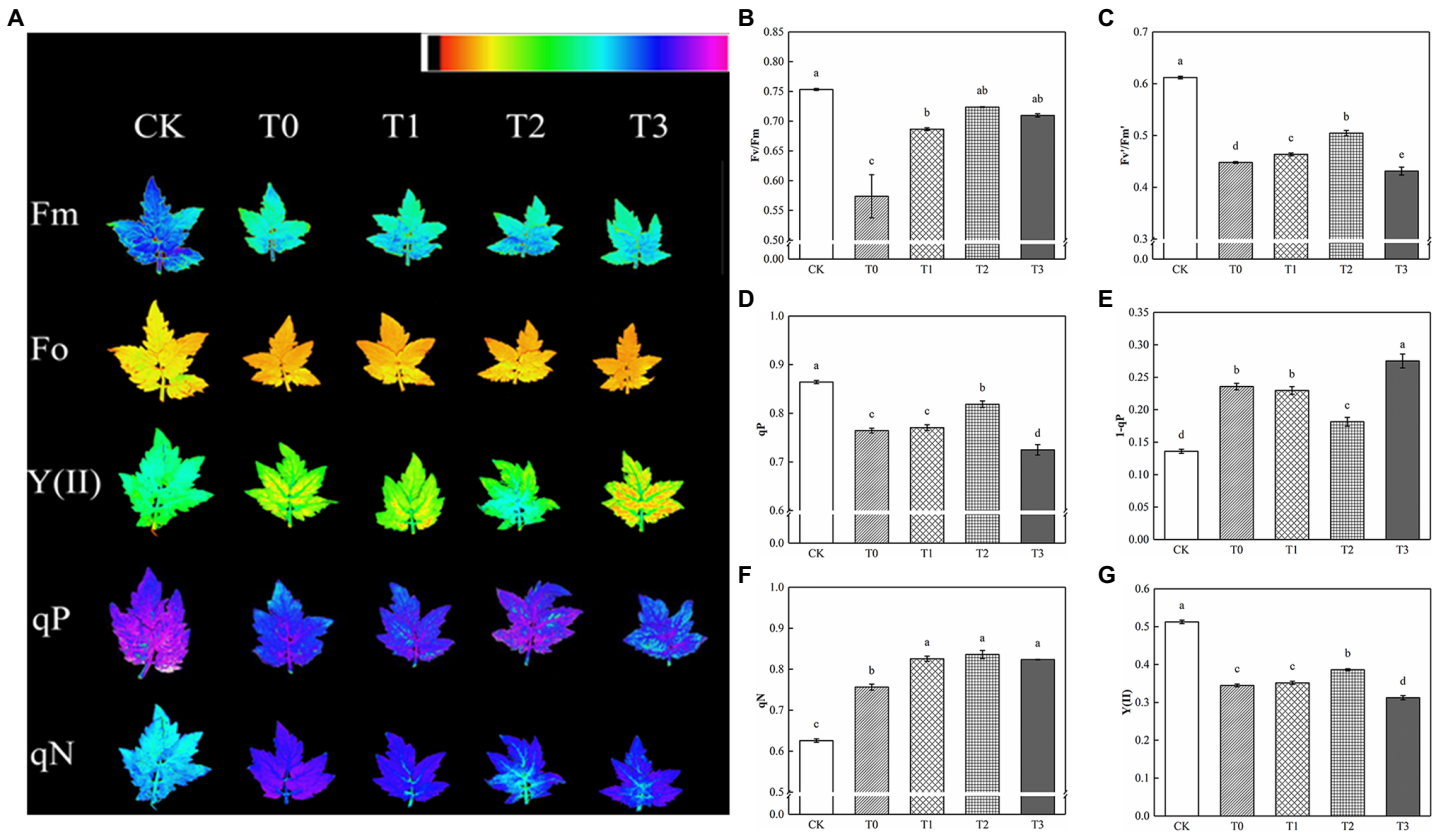
Effect of different concentrations of trehalose on the chlorophyll fluorescence parameters of tomato leaves. Data were recorded after 5 days of treatment. **(A)** Images of Fm, Fo, Y(II), qP and qN. These false colors are used to represent values of the parameter ranging from 0 (black) to 1.0 (purple). **(B)** Maximum efficiency of PSII photochemistry (Fv/Fm). **(C)** Efficiency of excitation energy captured by open PSII reaction centers (Fv′/Fm′). **(D)** Coefficient of photochemical quenching (qP). **(E)** PSII excitation pressure (1-qP). **(F)** Coefficient of non-photochemical quenching (qN). **(G)** Actual PSII efficiency (Y(II)). The results showed the mean ± SE of three replicates, and the different letters denote the significant difference among treatments (*p* < 0.05), according to Duncan’s multiple test. CK, control; T0, 150 mM NaCl; T1, 150 mM NaCl +5 mM trehalose; T2, 150 mM NaCl +10 mM trehalose; T3, 150 mM NaCl +25 mM trehalose.

Compared with CK, Fv/Fm and Fv′/Fm′ decreased significantly under salt stress, and the addition of exogenous trehalose alleviated the effect to varying degrees (Figure 3). No significant difference was observed between Fv/Fm of T2 and CK, and Fv′/Fm′ of T2 was remarkably higher than that of T1 and T3. Salt damage also resulted in the decline of qP and the increase of qN, which reduced the actual photochemical rate of PSII. Compared with T1 and T3, T2 drastically increased qP and Y(II) and decreased 1−qP. Therefore, 10 mM of trehalose could inhibit the excitation pressure of PSII in tomato plants, improve the distribution of absorbed energy caused by stress relief, and reduce excess energy.

**Figure 4:**
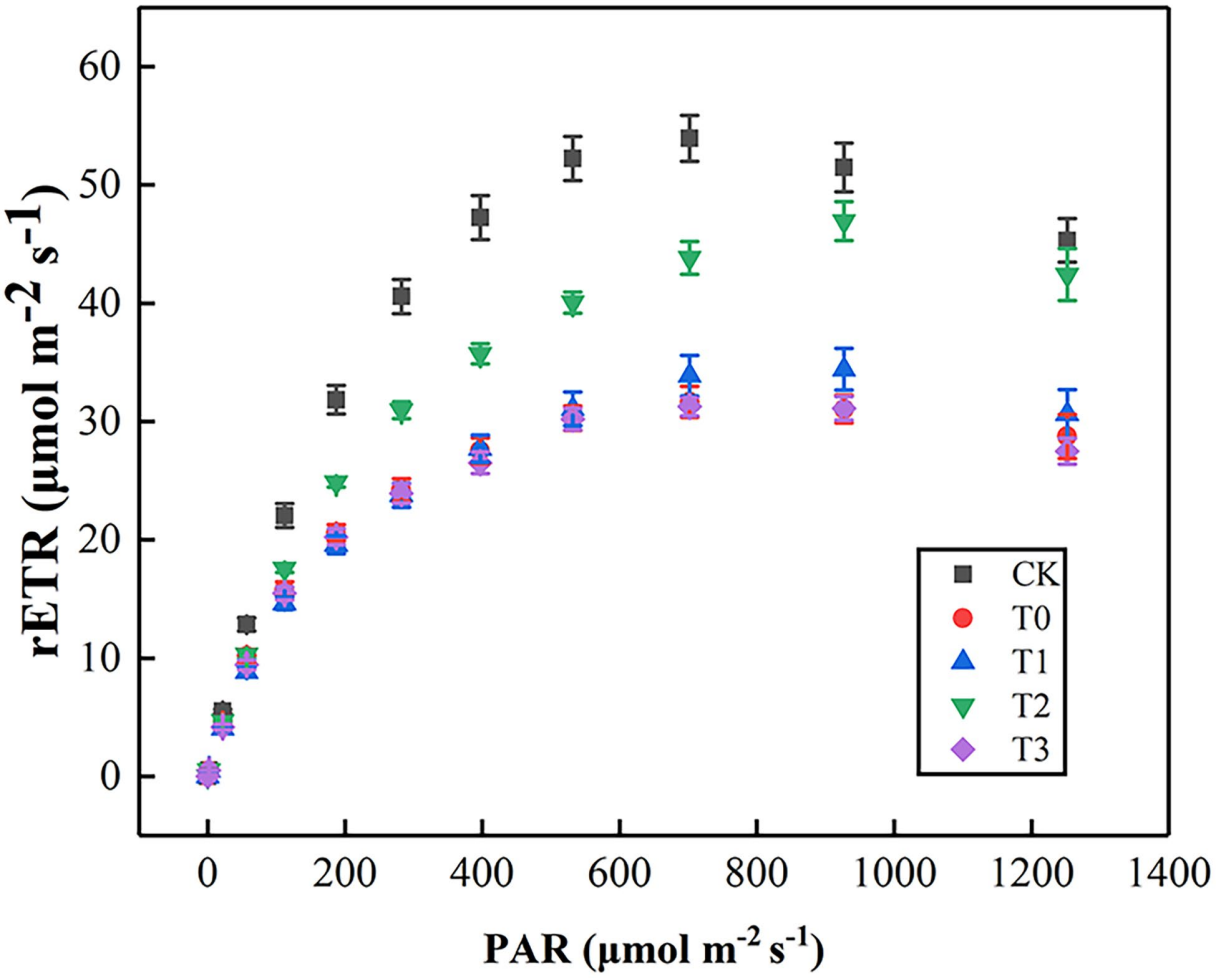
Effect of different concentrations of trehalose on the light response curve of tomato leaves. Data were recorded after 5 days of treatment. The results showed the mean ± SE of three replicates. CK, control; T0, 150 mM NaCl; T1, 150 mM NaCl +5 mM trehalose; T2, 150 mM NaCl +10 mM trehalose; T3, 150 mM NaCl +25 mM trehalose.

As shown in Figure 4, rETR initially increased, then slowed down and gradually stabilized with the enhancement of PAR. In comparison with CK, the rETR of T0 was significantly inhibited, but the value of T2 was always higher than other treatments. By fitting the light–response curves, we displayed that Pm, *α*, and Ik of T0 were lower than those of CK (Table 3). The inhibition effect of salt stress on Pm, *α*, and Ik in T2 were evidently alleviated. However, the low-concentration trehalose treatment (T1) had a weak alleviating effect, whereas the high-concentration trehalose treatment (T3) aggravated the damage of tomato leaves. Therefore, exogenous spraying of 10 mM of trehalose (T2) was the best mitigation concentration under salinity.

**Table 3:** Effect of different concentrations of trehalose on the fitting parameters of light–response curve in tomato leaves under saline conditions.

Treatments	rETRmax (μmol m^−2^ s^-1)^	*α*	IK (μmol m^−2^ s^−1^)
CK	97.481 ± 8.475a	0.235 ± 0.01a	438.849 ± 43.524a
T0	33.405 ± 1.889d	0.182 ± 0.007b	187.049 ± 9.104d
T1	41.096 ± 1.898cd	0.149 ± 0.006c	281.217 ± 12.81bc
T2	57.041 ± 4.456b	0.175 ± 0.006b	300.765 ± 30.793b
T3	35.459 ± 1.336d	0.169 ± 0.009bc	215.401 ± 12.177cd

Data were recorded after 5 days of treatment. Values (mean ± SE) were the average of three independent experiments. Values followed by a different lowercase letter in the same column indicated significant difference by Duncan’s multiple range test at *p* < 0.05. CK, control; T0, 150 mM NaCl; T1, 150 mM NaCl + 5 mM trehalose; T2, 150 mM NaCl + 10 mM trehalose; T3, 150 mM NaCl + 25 mM trehalose.

### Changes of Osmotic Substances Under Saline Conditions by Trehalose

**Figure 5:**
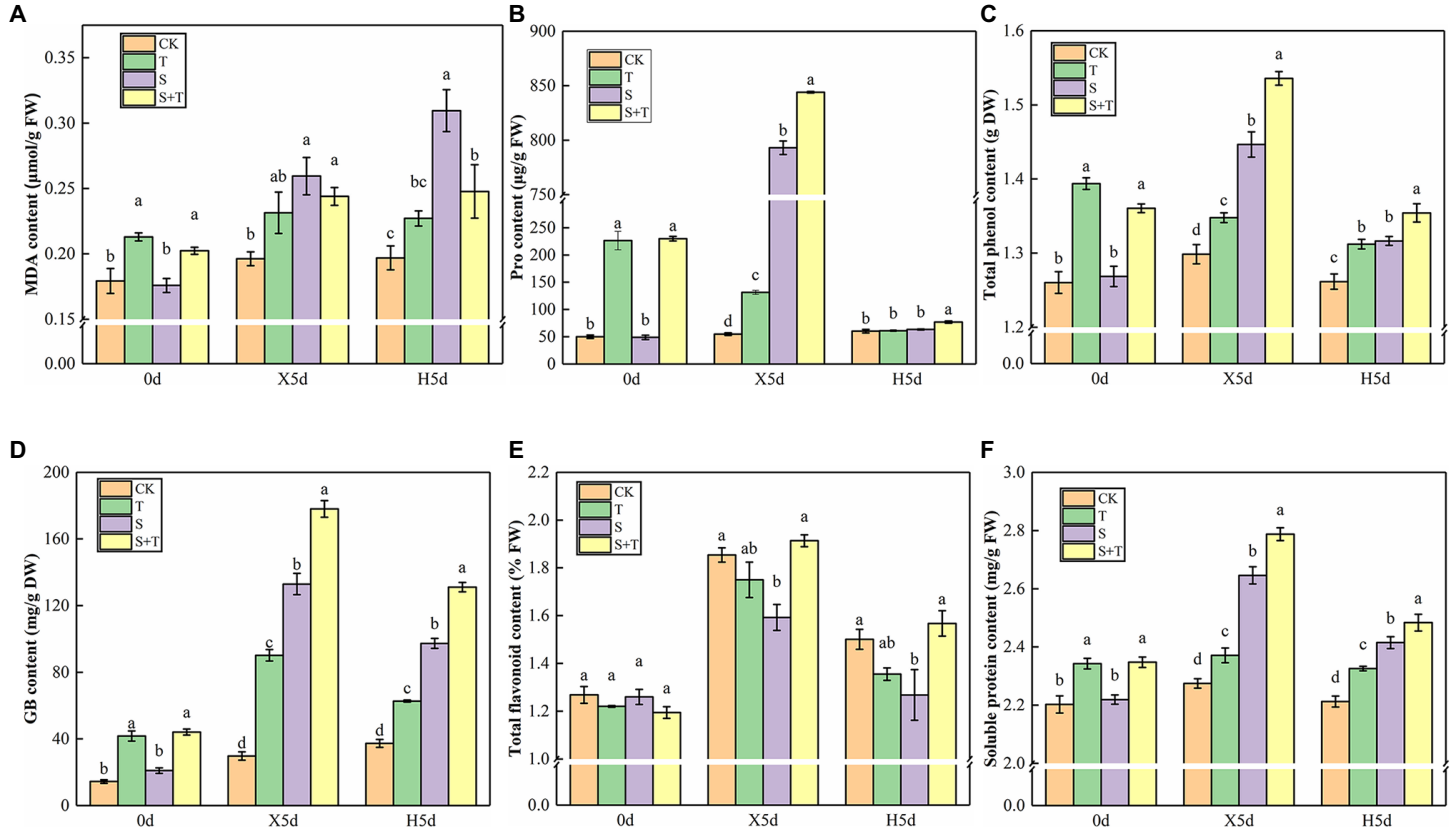
Impact of exogenously applied trehalose on osmotic substances of tomato leaves under stress and non-stress conditions. **(A)** MDA content. **(B)** Pro content. **(C)** Total phenol content. **(D)** GB content. **(E)** Total flavonoid content. **(F)** Soluble protein content. The results showed the mean ± SE of three replicates, and the different letters denote the significant difference among treatments (*p* < 0.05), according to Duncan’s multiple test. CK, control; T, 10 mM trehalose; S, 150 mM NaCl; S + T, 150 mM NaCl +10 mM trehalose.

We measured osmotic substances in tomato leaves to determine how trehalose affects damaged plants under salt stress (Figure 5). By comparison with CK, S increased drastically the contents of MDA, Pro, total flavonoids, GB, and soluble protein in tomato leaves and decreased the content of total phenol at 5d of salt stress (X5d). The foliar trehalose promoted the accumulation of Pro, total phenol, total flavonoids, GB, and soluble protein, whereas the accumulation of MDA was repressed. The contents of other substances, except for MDA, were diminished greatly, but S + T was still higher than S after recovery (H5d). The above-mentioned results suggested that trehalose could reduce the salt damage of tomato plants by regulating the contents of osmotic substances.

### Effects of Trehalose on Carbohydrate Contents and Trehalose Metabolism

**Figure 6:**
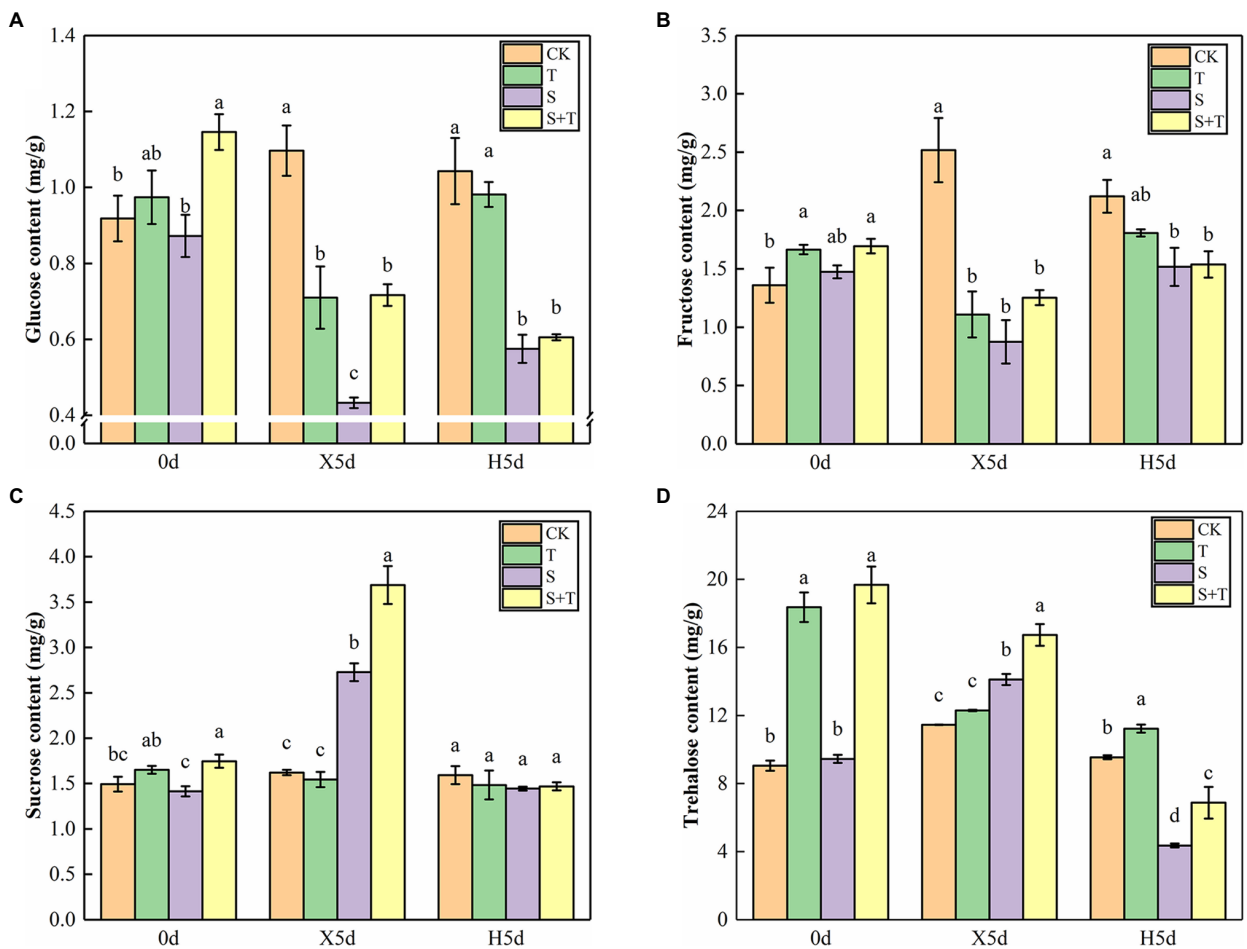
Effects of foliar-applied trehalose on carbohydrate content of tomato leaves under stress and non-stress conditions. **(A)** Glucose content. **(B)** Fructose content. **(C)** Sucrose content. **(D)** Trehalose content. The results showed the mean ± SE of three replicates, and the different letters denote the significant difference among treatments (*p* < 0.05), according to Duncan’s multiple test. CK, control; T, 10 mM trehalose; S, 150 mM NaCl; S + T, 150 mM NaCl +10 mM trehalose.

At 0 day, the contents of glucose, sucrose, fructose, and trehalose in tomato leaves increased after trehalose was applied (Figure 6). Under salt stress (X5d), the contents of sucrose and trehalose in S increased, and the effect of applying trehalose was more significant. In turn, the trends of fructose and glucose were not consistent with the two aforementioned carbohydrates. The application of NaCl alone significantly decreased the contents of glucose and fructose, and their contents increased evidently in the mixture treatment of NaCl and trehalose. After performing the restore operation (H5d), four types of carbohydrates in single or mixed salt treatment decreased markedly compared with CK, and the trehalose content in S + T was higher than the value of S.

**Figure 7:**
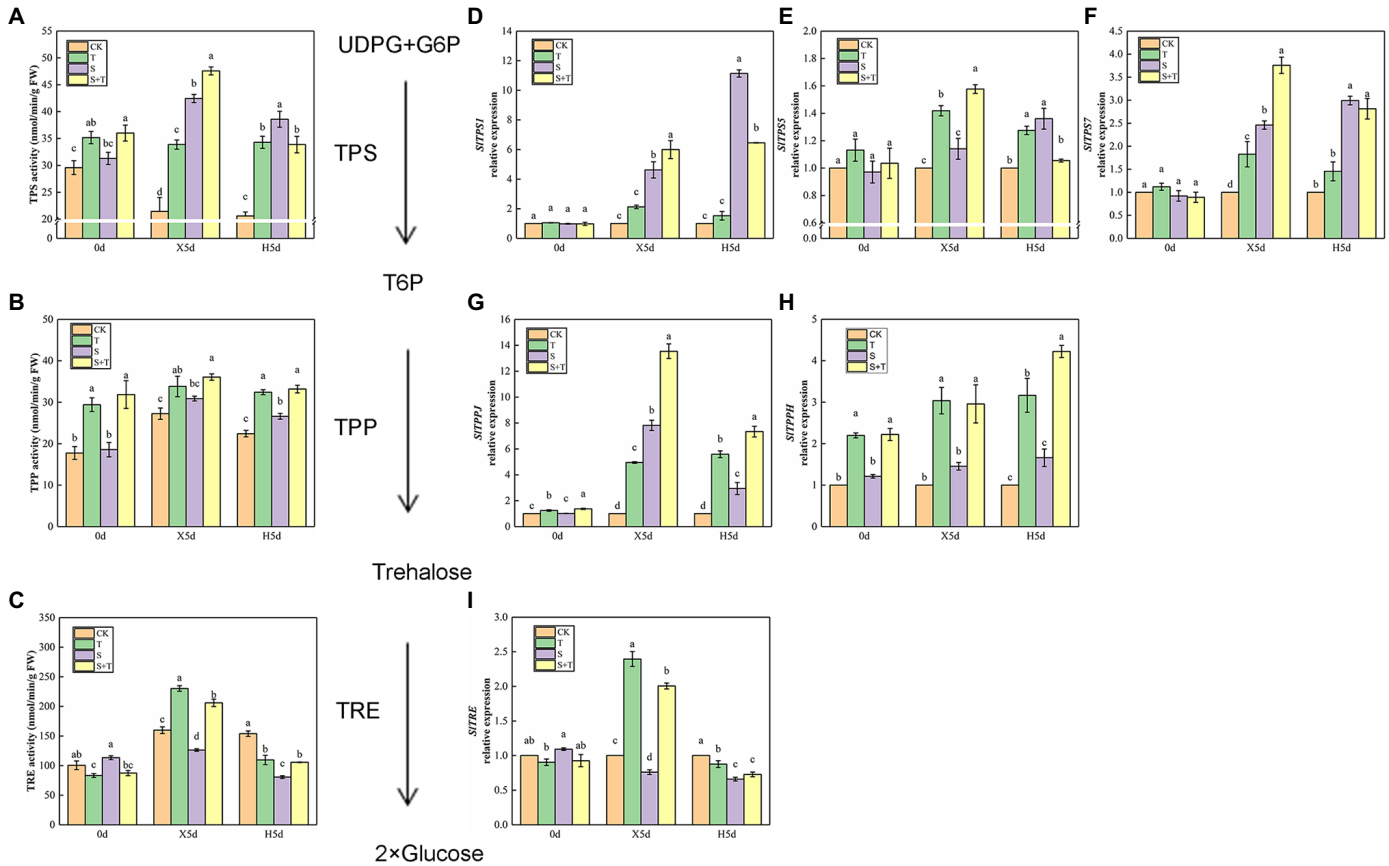
Impact of exogenously applied trehalose on trehalose metabolism of tomato leaves under stress and non-stress conditions. The TPS–TPP pathway was the only trehalose synthesis pathway in higher plants. First, uridine glucose diphosphate (UDPG) and glucose 6-phosphate (G6P) were catalyzed by trehalose-6-phosphate synthase (TPS) to form trehalose 6-phosphate (T6P). Second, T6P was catalyzed to trehalose by trehalose-6-phosphate phosphatase (TPP). Finally, trehalose was broken down by trehalase (TRE) into two molecules of glucose. **(A)–(C)** represent the activity of TPS, TPP, and TRE, respectively. **(D)–(I)** represent the expression levels of *SlTPS1*, *SlTPS5*, *SlTPS7*, *SlTPPJ*, *SlTPPH*, and *SlTRE*, respectively. The results showed the mean ± SE of three replicates, and the different letters denote the significant difference among treatments (*p* < 0.05), according to Duncan’s multiple test. CK, control; T, 10 mM trehalose; S, 150 mM NaCl; S + T, 150 mM NaCl +10 mM trehalose.

We detected the enzymatic activity of TPP, TPS, and TRE to explore the effect of trehalose on salt stress in tomato (Figures 7A–C). On 0 day, foliar-applied trehalose promoted the enzymatic activity of TPP and TPS but inhibited TRE activity. The variation in TPP and TPS activity of tomato seedlings was similar in salt stress (X5d) and recovery (H5d) periods, and S was higher than CK; in addition to the activity of TPS enzyme under the recovery period (H5d), we observed that the enzyme activity of S + T treatment was significantly higher than that of S treatment. Under salt stress conditions (X5d), in contrast to CK, salinity reduced the TRE activity in tomato, but it increased evidently after trehalose was applied. After recovery (H5d), the activity of TPP and TRE in S and S + T decreased slightly; nevertheless, the values of S + T were obviously higher than S.

Then, we examined trehalose-relative enzyme gene expression levels in tomato leaves (Figures 7D–I). We found that *SlTPS1*, *SlTPS5*, *SlTPS7*, *SlTPPJ* and *SlTPPH* genes were induced to express under saline conditions (X5d), and the upregulation trend was higher after the exogenous supply of trehalose. In addition, salt promoted the expression of *SlTPS1*, *SlTPS5* and *SlTPS7* during the recovery period (H5d), but this trend was reversed by the application of trehalose. The upregulation trend of *SlTPPJ* and *SlTPPH* genes continued under salt-free condition. Although the transcriptional level of *SlTRE* was inhibited by salinity (X5d), the application of trehalose stimulated its improvement. Compared with CK, the transcriptional level of *SlTRE* was downregulated in S and S + T during the recovery period (H5d). This phenomenon was consistent with the above-mentioned TRE enzymatic activity (Figure 7I). These results indicated that trehalose regulated carbohydrate contents and trehalose metabolism involved in trehalose-induced salt resistance.

### Temporal Regulation of ROS Contents, Antioxidant Enzyme, and Transcriptional Levels by Trehalose

**Figure 8:**
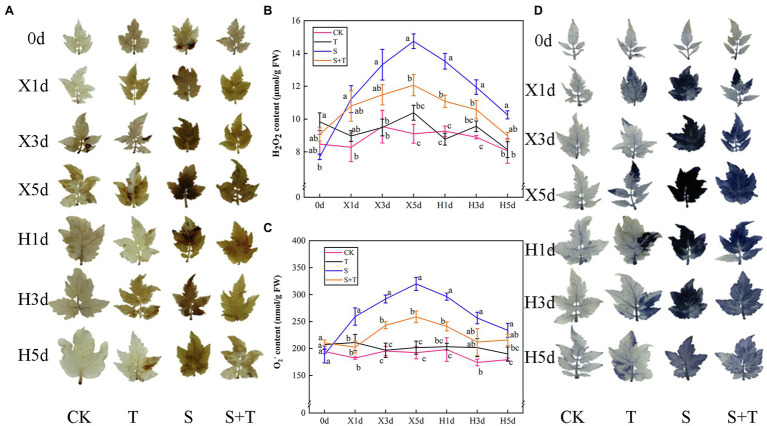
Effects of foliar-applied trehalose on ROS accumulation of tomato leaves under stress and non-stress conditions. **(A)** Diaminobenzidine (DAB) staining. **(B)** H_2_O_2_ content. **(C)** O_2_^−^ content. **(D)** Nitroblue tetrazolium (NBT) staining. The results showed the mean ± SE of three replicates, and the different letters denote the significant difference among treatments (*p* < 0.05), according to Duncan’s multiple test. CK, control; T, 10 mM trehalose; S, 150 mM NaCl; S + T, 150 mM NaCl +10 mM trehalose.

The accumulation of ROS in tomato leaves under different treatments is shown in Figure 8. NBT and DAB were used to visualize the contents of O_2_^−^ and H_2_O_2_ in tomato leaves. We investigated the contents of O_2_^−^ and H_2_O_2_of T more than CK. With the time-course extension of salt stress, the contents of O_2_^−^ and H_2_O_2_ were increased significantly by S. In addition, the intensity of dark-brown and blue staining was deeper. After the release of stress, the contents of O_2_^−^ and H_2_O_2_ in S and S + T decreased gradually, and salt stress has been dwindled to varying degrees. However, the application of trehalose affected distinctly the time course of ROS contents. For example, compared with S, the contents of two kinds of ROS in S + T were lower at the same time point, and the degree of staining was lighter.

**Figure 9:**
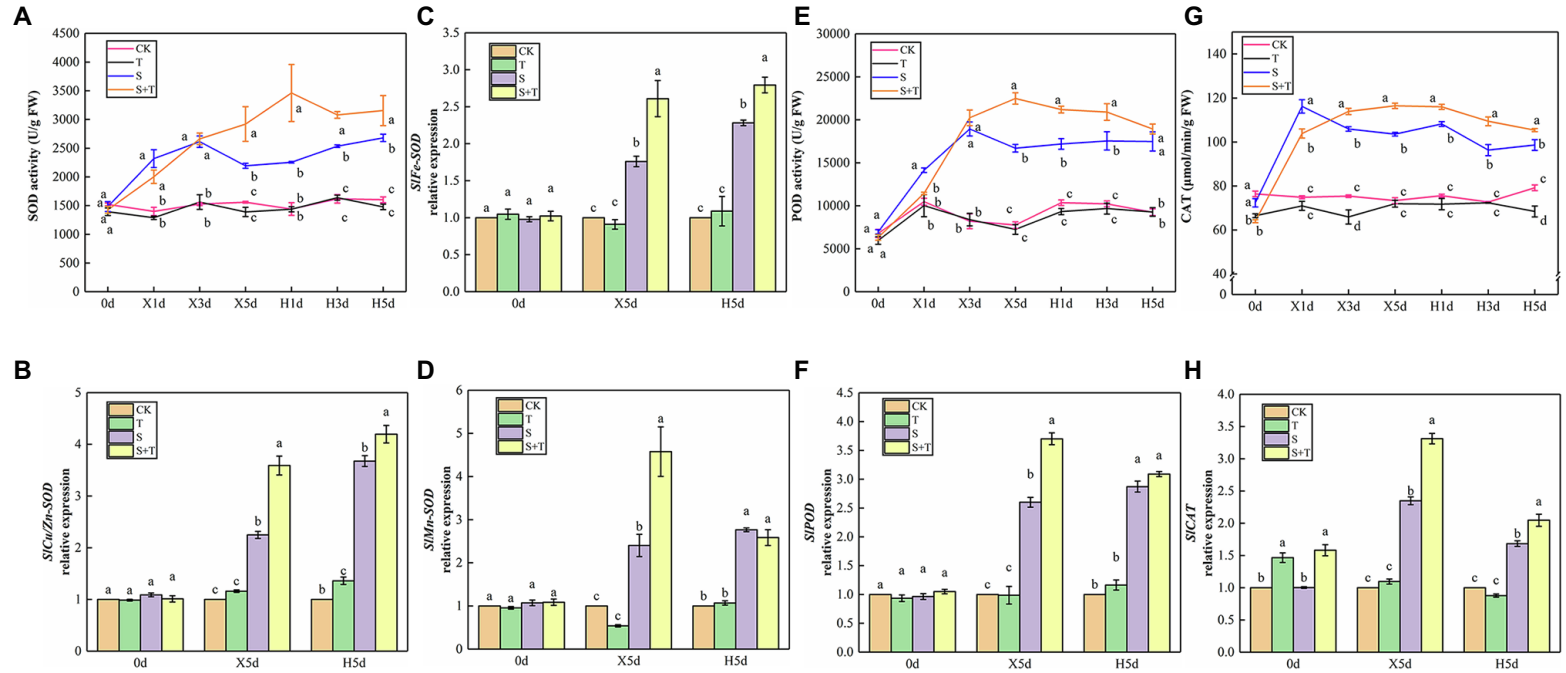
Impact of exogenously applied trehalose on ROS metabolism of tomato leaves under stress and non-stress conditions. **(A,E,G)** represent the activity of SOD, POD and CAT, respectively. **(B–D,F,H)** represent the expression levels of *SlCu/Zn-SOD*, *SlFe-SOD*, *SlMn-SOD*, *SlPOD*, and *SlCAT*, respectively. The results showed the mean ± SE of three replicates, and the different letters denote the significant difference among treatments (*p* < 0.05), according to Duncan’s multiple test. CK, control; T, 10 mM trehalose; S, 150 mM NaCl; S + T, 150 mM NaCl +10 mM trehalose.

By measuring and analyzing the antioxidant enzyme activity (SOD, POD, and CAT) of tomato leaves under salt and salt-free environments, we could further understand the mechanism of exogenous trehalose on the elimination of excessive accumulation of ROS caused by salt stress. As shown in Figures 9A,E,G, compared with the CK treatment, the activity of SOD and POD in T showed no significant discrepancy; the SOD, POD, and CAT activity of S increased rapidly and reached the peak from the beginning of salt stress (X1d or X3d) and then declined until reaching normal supply; the activity of POD and CAT showed a similar trend in S + T. Under the wipe-salt period, the activity of SOD and POD had a slow upward trend, whereas the activity of CAT decreased in S. Moreover, the activity of the three enzymes slightly decreased in S + T but still markedly higher than that in S.

The quantification of the expression levels of antioxidase-related genes was conducted in tomato leaves to support qualitative analysis (Figures 9B–D,F,H). We pointed out that application of trehalose alone upregulated *SlCAT* expression at 0 day. At 5 day after salt stress (X5d), the transcriptional levels of *SlCu/Zn-SOD*, *SlFe-SOD*, *SlMn-SOD*, *SlPOD* and *SlCAT* in tomato leaves sharply increased simultaneously, but the expression levels in S + T were always higher than that in S treatment. During the recovery period, the expression of *SlCu/Zn-SOD* and *SlFe-SOD* continued to increase, whereas the expression of other genes decreased. Meanwhile, compared with S, the transcriptional level of *SlCAT* was significantly upregulated in S + T, which was consistent with the CAT activity data in the above-mentioned experiments (Figure 9G). The qualitative and quantitative analyses of antioxidant enzyme in tomato leaves displayed that foliar-applied trehalose increased the activity of antioxidant enzymes and reduced the contents of ROS.

### Balance of Na^+^ and K^+^ Contents by Trehalose

**Figure 10:**
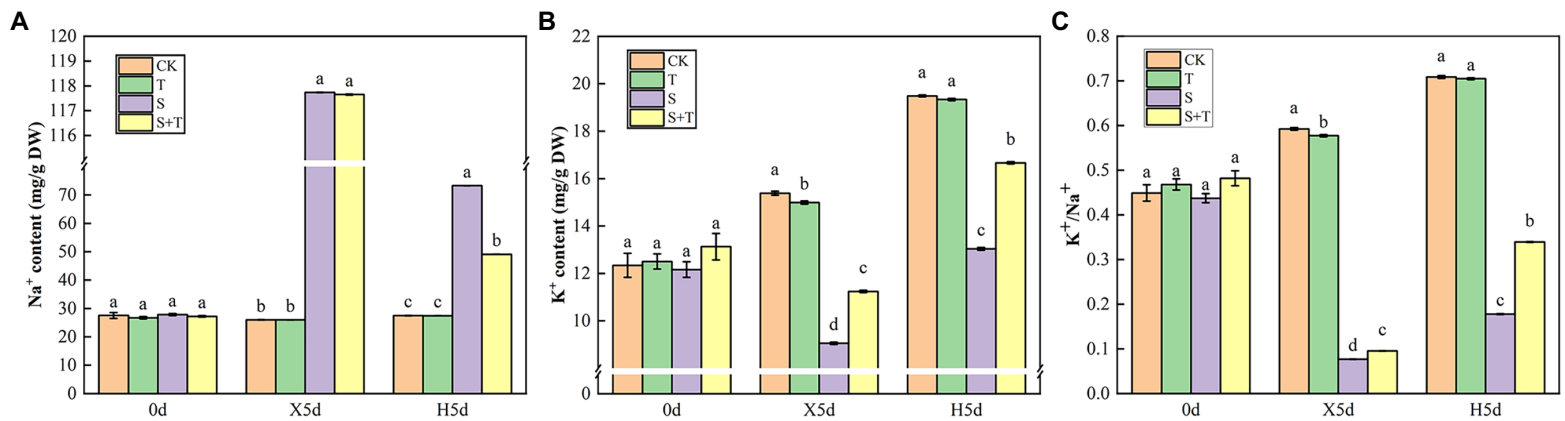
Effects of foliar-applied trehalose on ion content of tomato leaves under stress and non-stress conditions. **(A)** Na^+^ content. **(B)** K^+^ content. **(C)** K^+^/Na^+^ ratio. The results showed the mean ± SE of three replicates, and the different letters denote the significant difference among treatments (*p* < 0.05), according to Duncan’s multiple test. CK, control; T, 10 mM trehalose; S, 150 mM NaCl; S + T, 150 mM NaCl + 10 mM trehalose.

Mineral ions in tomato leaves were further measured as shown in Figure 10. At 0 day, there was no significant difference in ion content among the four treatments. Under salt stress (X5d), compared with the control, S treatment caused a 4-fold increase in the Na^+^ content in tomato leaves; however, it decreased the content of K^+^ and the ratio of K^+^/Na^+^ by 25.56% and 82.41%, respectively. Exogenous trehalose significantly increased content of K^+^ and the ratio of K^+^/Na^+^, but it had no critical influence on Na^+^ content. After performing the restore operation (H5d), the Na^+^ content was drastically reduced in S + T treatment compared with S treatment, while K^+^ content and K^+^/Na^+^ ratio were increased markedly. Consequently, the presence of exogenous trehalose significantly inhibited the accumulation of Na^+^ under salt stress and played a main role in maintaining K^+^/Na^+^ homeostasis in tomato plants.

## Discussion

### Relieving the Growth Inhibition

The effect of salt stress on plants primarily included osmotic stress and ion toxicity, which caused nutrient deficiency and breaking of energy balance (Wu et al., 2019; Wang et al., 2020c). Biomass is a comprehensive factor, which reflected salt stress in plants, and it is the direct index of salt resistance (Zhang et al., 2020c). In our case, we confirmed that with the increase of salt concentration, the inhibition degree of tomato seedlings was aggravated, and the cell damage rate increased (Figure 1). The osmotic effect caused by salt stress was related to the inhibition of cell wall extension and cell expansion (Shahzad et al., 2020). Thus, exogenous substances should be used to reverse the damage caused by salt stress on plants. The effect of trehalose on reducing the damage of tomato plants under salt stress was studied in our experiment. Our results clearly suggested that trehalose pretreatment promoted the growth and biomass of tomato seedlings under salt stress (Figure 2), which was consistent with the previous studies (Chang et al., 2014; Yang et al., 2014; Feng et al., 2019; Zhao et al., 2019; de Novais Portugal et al., 2021; Kosar et al., 2021). Of note, the role of trehalose in salt tolerance was dependent on the dose applied, and 10 mM was determined as the optimal concentration in this study. High concentrations of trehalose were supra-optimum, which adversely affected plant growth.

### Inhibiting Photosystem Damage

Chlorophyll fluorescence was used to detect the effects of any stress on photosynthesis. With regard to the chlorophyll fluorescence parameter, salt stress significantly reduced Y(II), Fv/Fm, and Fv′/Fm′ compared with the control in tomato plants (Figures 3B,C,G). Plants in adverse environments were subjected to PSII photoinhibition, which may be regulated by chronic photoinhibition and dynamic photoinhibition (Hikosaka, 2021). Therefore, the rapid decline of Fv/Fm was the result of chronic photoinhibition (Figure 3B), whereas the increase of Fv′/Fm′ and qN was caused by dynamic photoinhibition (Figures 3C,F). After the application of trehalose, fluorescence quenching was enhanced because of photosynthesis and heat dissipation (Figures 3D,F), which alleviated the imbalance between light energy absorption and metabolic consumption caused by salt stress to a certain extent, and reduced the excessive accumulation of excitation energy in the photosystem. Concurrently, 10 mM of trehalose could reduce the excitation pressure faced by PSII (Figure 3E), enhance the efficiency of excitation energy capture (Figure 3C) and inhibit the decrease of Y(II; Figure 3G). This result was similar to the findings in Lu et al. (2019). Moreover, Luo et al. (2010) showed that trehalose pretreatment protected the ultrastructure of chloroplasts and some peptides in thylakoids under heat stress. Zhao et al. (2019) indicated that leaves treated with trehalose had more intact chloroplasts. Therefore, we hypothesized that the thylakoid membrane structure was protected by the accumulation of trehalose or its metabolites, thereby improving the efficiency of light energy absorption, utilization, and distribution of tomato plants under NaCl stress. Notably, rapid light–response curve fitting was used to evaluate the potential photosynthetic capacity of plants under different light intensities (Ralph and Gademann, 2005). rETRmax can reflect the photosynthetic electron transport and phosphorylation activity of plant leaves (Coste et al., 2005). α and Ik reflected the light energy utilization efficiency and tolerance to hard light of plant leaves, respectively (Ralph and Gademann, 2005). This study found that rETRmax, α, and Ik of tomato leaves under salt stress were distinctly lower than those under normal conditions (Figure 4; Table 3), which was consistent with the results of Zhao et al. (2019) under drought conditions. 10 mM trehalose increased the maximum electron transfer rate (rETRmax), light energy utilization efficiency (α) and hard light resistance (Ik) of tomato plants under salt stress. Tang et al. (2021) also reached a similar conclusion in her study of zeaxanthin in alleviating chilling stress. Trehalose can relieve photoinhibition, which in turn will lead to positive effects on plant growth under saline conditions.

### Regulating Osmotic Substances

Plants will adopt defensive adaptation mechanisms such as osmotic regulation or osmoprotection to survive in adverse environments. MDA reflected the degree of lipid peroxidation in the cell membrane, which was closely related to the effective enzymatic clearance system in plants (Farooq et al., 2009). Previous studies have also shown that trehalose played a positive role in reducing MDA level in stress (Kosar et al., 2021). All of these studies suggested that excessive accumulation of MDA was a sign of membrane lipid peroxidation, which was generally low in resistant plants. Our data showed that the content of MDA increased significantly under salt stress, and S and T co-existed to inhibit the continued increase of MDA. Similarly, the MDA content of S + T was lower than S under the wipe-salt period (Figure 5A). Based on earlier studies, Pro and GB could play a role in regulating cell osmotic balance, scavenging reactive oxygen species and protecting membrane structure stability (Kumar et al., 2017). Phenol was important non-enzymatic antioxidants in plants because of their activity of stabilizing and delocalizing unpaired electrons as hydrogen or electron donors and their ability to chelate transition metal ions (Jaakola and Hohtola, 2010; Israr et al., 2011; Kleinwächter and Selmar, 2014; Nakabayashi et al., 2014; Sadak, 2019). Likewise, soluble protein was not only an osmotic regulator but also an important index to measure the damage of plant protein (Erice et al., 2010). These research results indicated that the contents of permeable substances responded to oxidative damage, and regulating the accumulation of osmosis was necessary to improve plant salt tolerance. In our experimental procedure system, foliar-applied trehalose promoted the accumulation of Pro, total flavonoids, total phenols, soluble protein, and GB under salt and salt-free environments (Figures 5B–F). These data were supported by the findings of de Novais Portugal et al. (2021), who observed that the effect of trehalose alleviated drought stress by increasing the contents of osmotic regulation substances. This effect may involve not only osmotic regulation but also other effects, such as membrane protection (Ahmad and Prasad, 2011). Collectively, we speculated that the unique physical properties of trehalose were associated with the accumulation of osmosis-regulating substances, but this hypothesis needed further investigation.

### Promoting Carbohydrate Contents and Regulating Trehalose Metabolism

Sugar metabolism was closely related to plant response to stress. Sugar could not only serve as the main carbon source for plant growth but also serve as a signal to respond to plant stress (Eveland and Jackson, 2012; Keunen et al., 2013). Sui et al. (2015) attributed that salt-tolerant sweet sorghum increased sucrose content by enhancing sucrose synthesis and reducing sucrose decomposition in salinity. Peng et al. (2016) and Nemati et al. (2018) extended that salt-tolerant cotton and drought-tolerant wheat seedlings were conducive to sucrose synthesis, improving plant energy storage and maintaining plant cell metabolism. Meanwhile, Siringam et al. (2012) demonstrated that exogenous sucrose could increase the contents of glucose and fructose of salt-sensitive rice under salt stress. Transcriptional analysis of *Arabidopsis* showed that glucose could induce the expression of a large number of stress-responsive genes (Price et al., 2004). In addition, Ambastha and Tiwari (2015) suggested that desert plants could gather high levels of trehalose in their bodies. By contrast, our study showed that salt stress increased significantly the contents of sucrose and trehalose, whereas the contents of glucose and fructose decreased (Figure 6). Therefore, we hypothesized that this phenomenon might be caused by the decreased hydrolysis rate of sucrose and the conversion rate of trehalose to glucose in tomato leaves under salt stress. Oszvald et al. (2018) found that high soluble carbohydrate levels could serve as a positive regulator of genes pertained to sugar sensing and carbon metabolism under saline conditions. In the present study, we show that foliar trehalose spraying promoted carbohydrate accumulation in tomato leaves under salt and wipe-salt period, including endogenous trehalose (Figure 6). These results were in line with Sadak (2019). Thus, the increase of trehalose content proved that trehalose improved the salt tolerance of tomato plants.

As an intermediate substance in trehalose metabolism, T6P was a signal substance indicating carbon source adequacy (Zhang et al., 2009). O’Hara et al. (2013) pointed out that T6P could promote growth when carbon supply was sufficient, but growth was hampered when the increase of T6P was not in balance with available carbon. Tsai and Gazzarrini (2014) and Smeekens (2015) regarded that T6P inhibited SnRK1 activity, thereby affecting the bZIP11 promoter associated with stress resistance, and the transcription of bZIPs was usually associated with changes in sucrose content. Based on our results (Figures 6D, 7), a positive correlation was observed between trehalose content and TPS enzymatic activity under salt stress. Meanwhile, compared with S, the activity of TPP enzyme in S + T treatment was evidently higher probably because the level of T6P in tomato leaves was strictly controlled by TPS and TPP-related activity to maintain the balance of the T6P:sucrose ratio (Nuccio et al., 2015). Moreover, Nuñez-Muñoz et al. (2021) found that the trehalose overexpressing lines exhibited an aberrant phenotype, given the signaling role of a T6P, which is derived from trehalose, this may be an adverse effect caused by the hyperaccumulation of trehalose. In this experiment, TRE activity was significantly increased by S + T treatment after recovery (Figure 7C). It was speculated that the trehalose content in plants was controlled by altering trehalase activity and the toxicity of trehalose in plants was avoided under normal conditions. Alternatively, trehalose treatment could activate the ABA signaling pathway and alleviate drought stress (Yu et al., 2019). Subsequently, Wang et al. (2020b) and Lin et al. (2020) verified that ABA promoted the expression of *AtTPPE* and *AtTPPI* through ABF2, thereby regulating the expression of oxidative stress response genes. In summary, trehalose is implicated as an inducer or signal molecules that interacts with other signaling pathways, and these interactions may also have a significant effect on the plant’s own trehalose metabolism. However, little is known about the underlying mechanisms and physiological significance of these reactions. Understanding these interactions appears to be a fertile area for future research, potentially offering ways to improve crop plant defense against abiotic stresses.

### Alleviating Oxidative Stress

ROS was byproducts of aerobic metabolism (Hasanuzzaman et al., 2020). Many physiological functions were involved in the constitutive production of controlled ROS levels. Appropriate amount of oxygen radicals could be used as signaling molecules to regulate plant growth, hormone activities, transcription factor activities, and so on (Damiano et al., 2020). Hou et al. (2021) found that the membrane damage from accumulation of ROS under chilling stress. Excessive ROS also resulted in a significantly higher degree of oxidative damage under high Pb and Cd stress (Zhang et al., 2020a). Moreover, ROS levels were stable under normal conditions; but the outbreak of ROS occurred under salt stress (Zhao et al., 2021). Similarly, Akyol et al. (2020) showed that salt stress-induced ROS production, which led to disruption of important cellular functions in plants. The results of this study showed that with the time extension of salt stress, the content of O_2_^−^ and H_2_O_2_ in tomato leaves maintained higher levels. As shown by DAB and NBT staining, the tissue expression patterns of tomato leaves were similar to the content of H_2_O_2_ and O_2_^−^ (Figure 8). The ROS content was controlled not only by production but also by scavenging mechanism. Antioxidant enzymes and non-enzymatic antioxidants were the major scavenging forces of ROS and were essential for stress resistance in plants (Dvořák et al., 2021). SOD, CAT, and POD were the main ROS-removing enzymes in plants (Zhao et al., 2020). Zhang et al. (2020a) assumed that the increase of ROS contents stimulated the increase of SOD, POD, CAT, and other enzyme activities under adverse conditions. In addition, Xie et al. (2019) observed that the salt-tolerant wheat displayed reduced accumulations of H_2_O_2_ and higher activities of CAT, POD, and APX than salt-sensitive cultivars in virtue of reduced oxidative damage. Based on the above evidence, the high expression level of enzymatic antioxidants induced by salinity revealed an effective way to reduce Na^+^ toxicity. In our study, we also detected that the activity of antioxidant enzyme increased in the early stage of stress, which might be related to the activation of plant defense mechanism under salt stress; however, decrease in antioxidant enzyme activity at late stress stage might be due to the disruption of ROS homeostasis, which led to the increase of H_2_O_2_ and O_2_^−^ contents (Farooq et al., 2016; Figure 9). Foliar-applied trehalose further increased antioxidant enzyme activity under salt stress, the upregulation of their genes was noted with the same pattern, and the ROS level was significantly lower than the untreated plants (Figure 9). This suggested that trehalose was involved in enhancing the detoxification effect of ROS, reducing cellular damage and death, and alleviating salinity induced inhibition. Similar effects of trehalose on antioxidant enzyme activity have also been observed in plants under different stresses (Aldesuquy and Ghanem, 2015; Zhao et al., 2019; Liu et al., 2020). Total phenol, total flavonoids, Pro and GB acted not only as osmotic substances but also as non-enzymatic antioxidants (Xie et al., 2019), and the accumulate of these provided the reducing power to maintain the redox state and ROS homeostasis (Zhang et al., 2020b). In addition to enhancing the enzymatic antioxidant system, trehalose pretreatment tomato seedlings also increased the antioxidant contents (Figure 5). Interestingly, the antioxidant enzyme activity of S + T treatment decreased slightly, but it was still higher than that of S treatment under the recovery period (Figure 9). We hypothesized that the continuous effect of trehalose on antioxidant enzyme activity may be one of the mechanisms of plant tolerance to NaCl-induced oxidative stress in tomato seedlings. In addition, Zhang et al. (2020b) pointed out that cysteine sulphur in protein was the main target of H_2_O_2_-dependent oxidation, which might regulate ROS contents and apoplastic redox state. In previous studies, Krasensky et al. (2014) showed trehalose phosphate synthase (*AtTPPD*) had two cysteine residues 159 and 235, and could form intramolecular disulfide bonds under the oxidation condition, which seemed to be partly the reason why trehalose could affect the accumulation of ROS under salt stress and reduce oxidative damage. Unfortunately, the exact mechanism which trehalose specifically regulated redox signals remained unclear.

### Maintaining Ion Homeostasis

Sodium was not an essential element for plant growth and development, and excessive accumulation could cause plant poisoning (Yang et al., 2021). The reestablishment of ion homeostasis was one of the crucial mechanisms by which plants enhance salt tolerance (Guo et al., 2020). Indeed, it has been reported that salt stress increased Na^+^ concentration, but decreased K^+^ concentration and K^+^/Na^+^ ratio in tomato (Bhattarai et al., 2021), cotton (Guo et al., 2019) and wheat (Javaid et al., 2019). A similar phenomenon was found in our study. This might be due to the similar ionic radii and hydration of K^+^ and Na^+^, which had competitive inhibitory effects on plant cell entry (Li et al., 2017). Once excess Na^+^ entered plant cells, it directly damaged the cell wall, disrupted cell metabolism, which in turn triggered K^+^ efflux (Zamani et al., 2019). K^+^ played a key role in plant alleviating abiotic stresses, such as protein synthesis, charge balance, enzyme activation, and solute transport (Wu et al., 2018; Zhu et al., 2020). In several reports, it was assumed that the tolerant varieties had better K^+^ retention ability than the sensitive counterparts, such as barley (Wu et al., 2013) and wheat (Wu et al., 2015). Therefore, salt-stressed plants could suffer from both Na^+^ toxicity and low K^+^ concentration (Javaid et al., 2019). Guo et al. (2020) believed that maintaining a relatively high K^+^/Na^+^ ratio in tissues was more primary than retaining a lower Na^+^ concentration alone. Although there was no significant difference in Na^+^ concentration among the roots of salt-tolerant and salt-sensitive cotton, the K^+^/Na^+^ ratio of salt-tolerant roots was higher than that of salt-sensitive roots (Guo et al., 2019). Furthermore, Tang et al. (2019) investigated that Na^+^ extrusion was a pivotal mechanism to maintain a low Na^+^ concentration and high K^+^/Na^+^ ratio under salt stress, thus achieving salt tolerance. In a previous study, trehalose pretreatment was found to reduce Na^+^ accumulation, increase potassium content, and increase K^+^/Na^+^ ratio in *Catharanthus roseus* (Chang et al., 2014), which was consistent with our results. In our experimental procedure system, compared with the salt period, Na^+^ contents of S and S + T treatment under wipe-salt period decreased by 37.76% and 58.26%, respectively; K^+^ contents increased by 30.54% and 32.53%, respectively (Figure 10). These results demonstrated that trehalose on tomato plant response to salt stress was mainly by reducing the accumulation of Na^+^ and K^+^/Na^+^ ratio reduction, a possible reason was that trehalose promoted the efflux of Na^+^ and/or increased the vacuole isolation of Na^+^ by preserving the integrity of the protein and lipid bilayer, but the regulation of the plant ion balance mechanism still needed further research.

## Conclusion

The NaCl stress environment makes tomato plants passively absorb a large amount of Na^+^, which breaks the ion balance of the cell itself. Foliar application of trehalose could regulate ROS metabolism, photosynthesis, osmolyte synthesis, ion contents, and trehalose metabolic pathways, reduce the ion toxicity of tomato seedlings and ensure the normal physiological activities of cells. Although this study could provide comprehensive understanding of the mechanisms through which trehalose affects the tomato plant under saline conditions, the molecular mechanisms remain unknown. Thus, further studies on the mechanisms of trehalose in tomato salt tolerance should focus on protein modifications and signal interactions.

## Data Availability Statement

The datasets presented in this study can be found in online repositories. The names of the repository/repositories and accession number(s) can be found in the article/supplementary material.

## Author Contributions

YYang and JX conceived and designed the research. YYang, YYang, DD, and JZ conducted the experiments. LH and JL analyzed the data and prepared the figures and illustrations. YYan wrote the manuscript. PB and XZ read the manuscript and made valuable inputs. All authors contributed to the article and approved the submitted version.

## Funding

This research was supported by National Natural Science Foundation of China (32072657), China; the Special Fund for Technical System of Melon and Vegetable Industry of Gansu Province (GARS-GC-1), China; the National Key Research and Development Program of China (2016YFD0201005), China and the Outstanding Graduate Student “Innovation Star” Project of Gansu Province (2021CXZX-376), China.

## Conflict of Interest

The authors declare that the research was conducted in the absence of any commercial or financial relationships that could be construed as a potential conflict of interest.

## Publisher’s Note

All claims expressed in this article are solely those of the authors and do not necessarily represent those of their affiliated organizations, or those of the publisher, the editors and the reviewers. Any product that may be evaluated in this article, or claim that may be made by its manufacturer, is not guaranteed or endorsed by the publisher.

## Abbreviations

ROS: Reactive oxygen species
NaCl: Sodium chloride
REC: Relative electrical conductivity
Fv/Fm: Maximum efficiency of PSII photochemistry
Fv′/Fm′: Efficiency of excitation energy captured by open PSII reaction centers
qP: Coefficient of photochemical quenching
1-qP: PSII excitation pressure
qN: Coefficient of non-photochemical quenching
Y(II): Actual PSII efficiency
PAR: Photosynthetically active radiation intensity
rETR: Relative electron transfer rate
rETRmax: The maximum relative electron transfer rate
Ik: Half saturation and light intensity
*α*: The initial slope of the rapid light curve
β: The photoinhibition parameter
Pro: Free proline
MDA: Malondialdehyde
GB: Glycine betaine
H_2_O_2_: Hydrogen peroxide.
O_2_^−^: Superoxide anion.
SOD: Superoxide dismutase
POD: Peroxidase
CAT: Catalase
T6P: Trehalose 6-phosphate
TPP: Trehalose-6-phosphate phosphatase
TPS: Trehalose-6-phosphate synthase
TRE: Trehalase
